# A Disposable Screen Printed Electrodes with Hexagonal Ni(OH)_2_ Nanoplates Embedded Chitosan Layer for the Detection of Depression Biomarker

**DOI:** 10.3390/mi14010146

**Published:** 2023-01-05

**Authors:** Satyanarayana Moru, Venishetty Sunil Kumar, Shekar Kummari, Kotagiri Yugender Goud

**Affiliations:** 1 Department of Chemistry, School of Advanced Sciences, VIT-AP University, Amaravati 522237, India; 2 Department of Physical Sciences, Kakatiya Institute of Technology & Science, Warangal 506015, India; 3 Department of Chemistry, Indian Institute of Technology Palakkad, Palakkad 678 557, India; 4 Institute of Nanobiotechnology, Johns Hopkins University, Baltimore, MD 21218, USA

**Keywords:** point-of-care diagnosis, electrochemical detection, depression biomarker, serotonin, screen printed sensor, Ni(OH)_2_ nanoplates

## Abstract

Serotonin (5-hydroxytryptamine (5-HT)) is one of the important neurotransmitters which is released from the endocrine system. An abnormal level of this biomarker leads to several neurological diseases. The accurate assessment of serotonin is the utmost option to start treatment in the early stages of the disease. The current work is focused on the development of a disposable, screen-printed electrochemical sensor for the depression biomarker, serotonin in the physiological pH medium (pH 7.4) with the aid of a hexagonal, Ni(OH)_2_-nanoplate (NH-HNP)-embedded chitosan (Chit) and modified, screen-printed carbon electrode (SPCE). Initially, hexagonal nanoplates of Ni(OH)_2_ were synthesized by an eco-friendly and simple hydrothermal method. The prepared materials were well characterized by advanced analytical techniques to examine the physicochemical properties of the synthesized Ni(OH)_2_ hexagonal nanoplates. From the cyclic voltametric (CV) analysis, it was found that the oxidative current response of 5-HT at a NH-HNP-modified SPCE has about fivefold higher current values than over bare SPCE. The scan rate studies of NH-HNP-Chit/SPCE electrodes revealed that the oxidation mechanism of 5-HT is controlled by the diffusion behavior of the analyte. Differential pulse voltammetric tests of the NH-HNP-Chit/SPCE electrode exhibited a linear response in the dynamic concentration range of 0.1 to 30 µM, with a detection limit of about 60 nM. The sensor response is very reproducible from electrode to electrode, and the deactivation or surface-fouling of the sensor was not observed within the several experimental measurements. The sensor exhibited excellent storage stability over a period of twenty days. Finally, the fabricated, disposable SPCE sensor has shown respectable activity for the detection of depression biomarker 5-HT from synthetic urine and saliva samples.

## 1. Introduction

Neurotransmitters play a major role in communication within the neuron network through chemical signaling, which leads to human emotions. Several neurological disorders, such as dopamine, serotonin, etc., are associated with the concentration levels of these neurotransmitters. The easily oxidizable nature of these neurotransmitters via electrochemical techniques provides unique information about neurological disorder diagnoses [1]. Human emotional functions such as humor, sleep, sexuality, and enthusiasm are controlled by the vital neurotransmitter called serotonin (5-hydroxytryptamine or 5-HT), combined with other neurotransmitters such as dopamine and epinephrine. Researchers have discovered that there are a large number of people worldwide who suffer from neurological diseases, such as Parkinson’s and Alzheimer’s, which are caused by a deficiency of neurotransmitters, primarily serotonin [2,3,4]. Apart from this, 5-HT deficiency has been linked to a variety of disorders, most notably depression, migraine, bipolar disorder, and anxiety. Their coexistence in biological systems, as well as their mutual influence on each other’s activities, has been described in a significant number of publications. Exploration of the physiological quantities of 5-HT is therefore critical for elucidating specific biological functions [5,6].

The quantitative detection of 5-HT has been demonstrated using a variety of analytical techniques, including chromatographic methods [7,8], mass spectroscopy [9], chemiluminescence [10], and spectrophotometry [11], among others. However, the above-mentioned analytical techniques have some limitations, such as requiring a higher time of analysis, expensive instrumentation, complexity in sample preparation and calibration for quantitative analysis. These difficulties certainly make them incompatible for point-of-care analysis. Alternatively, electrochemical methods can overcome these difficulties because they are inexpensive, have a high sensitivity, portable instrumentation, and require much less measurement time [12]. However, the electrochemical measurements also suffer from separating the interfering molecules for the accurate detection of neurotransmitter concentrations. Dopamine is a commonly interfering molecule with concentrations higher than the serotonin. For this reason, it is important to pay attention to the development of selective and sensitive sensor systems to resolve these problems. Thus, the majority of researchers are working on developing the nanomolar quantities and precise electrochemical detection methodologies for 5-HT by tuning the sensor-interface materials’ properties. 

Use of transition-metals-based redox mediators in electrochemical sensors has been increasing due to their electrocatalytic nature in various electrochemical reactions. Among transition metals, nickel-based nanomaterials have a vital importance as electrocatalytic materials due to their high redox-active behavior. The oxide and hydroxide of nickel (II) are the most stable of the various forms of nickel (II) in air and solution, respectively [13,14]. At its nanocrystalline size, the electrocatalytic properties are further improved. To obtain the nanostructure of nickel hydroxide and oxide-based materials, many synthetic strategies have been reported [15]. Among them, the hydrothermal method is advantageous since it requires very limited and inexpensive raw materials and equipment, and there is no necessity of harmful organic solvents [16,17,18]. Additionally, the hydrothermal process is simple and produces materials with extremely selective phases, homogeneous distribution, and specific morphologies [19]. Generally, nickel hydroxide exists in two major polymorphs; these are α-Ni(OH)_2_ and β-Ni(OH)_2_. From the electrochemical-activity point of view, the α-Ni(OH)_2_ form possesses the higher electrochemical activity. However, it demonstrates less stability in the α-Ni(OH)_2_ phase and rapidly converts to the β-Ni(OH)_2_ phase, making it imperfect for electrochemical applications [20,21]. Consequently, the β-Ni(OH)_2_ form is considered to be a worthy material for an electrocatalyst. Additionally, the activity of Ni(OH)_2_ is also subject to various other features, such as active surface area, crystallite size, type of microstructure, and redox behavior, etc. [13]. Considering these factors, in this study we aimed to synthesize nanostructured β-Ni(OH)_2_ using the hydrothermal process, and examined its electrocatalytic property toward sensitive and selective quantification of 5-HT using a disposable, screen-printed carbon electrode (SPCE).

One of the critical steps in developing sensitive and long-term active electrochemical sensors is the formation of a stable, electrocatalytic conducting film on the surface of the electrode using an active material. Generally, polysaccharide-based biopolymers are considered to be the finest material for making the stable sensors layer without impeding the activity of the electrocatalytic material. Chitosan (Chit) is a biopolymer made from the deacetylation of chitin, an essential component of crab and shrimp exoskeletons and fungi cell walls [22,23]. It has merits such as a strong and uniform layer-formation property, demonstrated by embedding other materials, and also it shows biocompatibility with strong bonding and mechanical strengths [23,24]. With these advantages, chitosan was chosen in the present work as a vital component for dispersing and binding the Ni(OH)_2_ active material to form the stable and uniform sensing layer on the SPCE surface as recognition elements. All of these synergistic factors contribute to the NH-HNP-Chit/SPCE sensor’s high responsiveness, large detection range, durability, and accurate results in the detection of 5-HT. 

## 2. Experimental

### 2.1. Materials

Ni(NO3)_2_·6H_2_O(AR) and chitosan with a low molecular weight (60–120 KDa, 85% deacetylation) were obtained from Sigma Aldrich, Waltham, MA, USA. Serotonin hydrochloride (99% purity) was procured from Acros. The phosphate buffer solution (PBS) of 50 mM was prepared using Na_2_HPO_4_, NaH_2_PO_4_, and NaCl, and the solution pH was adjusted to 7.4 by using 0.1 M of NaOH. The stock solution of 1 mM 5-HT was prepared using a 50 mM PBS (pH 7.4) solution. The composition of the artificial urine and saliva is included in Table S1 (Supplementary Materials).

### 2.2. Preparation of Ni(OH)_2_ Hexagonal Nanoplates

**Figure 1:**
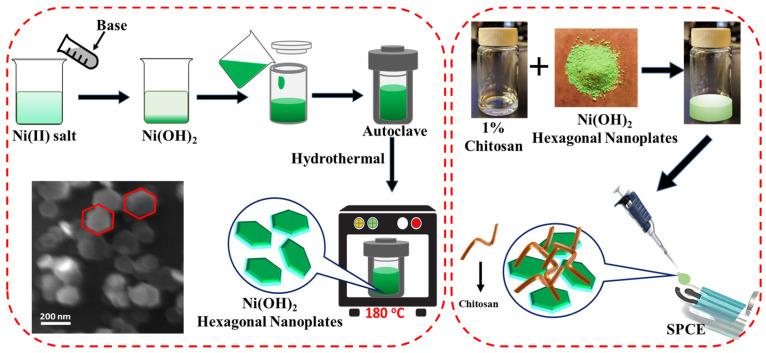
Schematic representation of hydrothermal synthesis process of the Ni(OH)_2_ hexagonal nanoplates and the NH-HNP-Chit-modified SPCE fabrication.

Hexagonal nanoplates of nickel hydroxide (NH-HNP) were synthesized using Ni(NO_3_)_2_·6H_2_O(AR) and NaOH(AR) by adopting the hydrothermal process. The detailed synthesis process is represented in the schematic diagram (Figure 1). The aqueous precursor solutions of the Ni(NO_3_)_2_ 6H_2_O (2.5 mmol) and sodium hydroxide (5 mmol) solutions were prepared separately. The two above-mentioned aqueous solutions were quickly mixed together and stirred for 15 min. The suspension mixture was later dispensed to a 60 mL Teflon container. The mixture was transferred to a stainless-steel autoclave and tightly sealed, after which it was programmed to 180 °C and left for approximately 4 hrs to complete the reaction under high-pressure conditions. Later, the precipitation mixture was centrifuged and washed several times with ethanol and deionized water. The resultant pure Ni(OH)_2_ nanoplates were collected. Finally, the nanoplates were dried in an oven at a temperature of 80 °C. The above-mentioned procedure yielded uniform Ni(OH)_2_ hexagonal nanoplates.

The structure and phase analysis were carried out using X-ray Diffraction (XRD, Brucker AXS D8 diffractometer). Morphological studies and crystal size were perceived using a field-emission-scanning electron microscope (FESEM, TESCAN-MIRA3 LM). X-ray photoelectron spectroscopy (XPS) measurements were carried out using an Omicron Nanotechnology instrument with Mg Kα (*hν* = 1253.6 eV) radiation.

### 2.3. Preparation of NH-HNP-Chit/SPCE

To begin, an electrochemical pre-activation of SPCE was performed using cyclic voltametric scanning in 0.5 M of H_2_SO_4_ at a potential range of 1.0 to 1.5 V and a scan rate of 100 mV s^−1^ for 6 cycles in order to acquire the freshly activated surface of SPCE. A 1% chitosan solution was prepared by dissolving 1 g of chitosan powder in 100 mL of acetic acid solution. Later, desired amounts (2, 4, and 6 mg) of Ni(OH)_2_-hexagonal-nanoplate powder were dissolved in 1 mL of chitosan solution and sonicated for 1 h. The final obtained solution mixtures were denoted as 2 wt% NH-HNP-Chit, 4 wt% -NH-HNP-Chit, and 6 wt% NH-HNP-Chit, respectively. The homogeneous suspensions were drop-casted onto electrochemically activated SPCE and allowed to stand at room temperature for an overnight period of time (Figure 1). The electrodes that resulted are denoted as 2 wt% NH-HNP-Chit/SPCE, 4 wt% NH-HNP-Chit/SPCE, and 6 wt% NH-HNP-Chit/SPCE, respectively. 

### 2.4. Electrochemical Analysis

Electrochemical measurements were conducted using an EmStat3 Blue wireless potentiostat. The screen-printed carbon electrodes (SPCEs) were fabricated using the DEK 248 screen-printing system. These SPCEs were printed with three electrodes consisting of graphite in disk shape as a working electrode (4 mm diameter), graphite in a curved line shape as a counter, and Ag/AgCl ink in a straight-line shape as a reference electrode (Figure 1). All electrochemical tests were conducted with a modified SPCE strip acting as a disposable electrode configuration, and 50–100 µL of test sample solution was administered to the sensor strip to cover all three electrodes. The DPV experiments were conducted by using the following parameters: 200 ms pulse width, 0.005 V pulse increment, 0 V standby potential, 100 mV pulse amplitude, and a potential scan range from 0.0 V to 0.5 V. 

## 3. Results and Discussion 

### 3.1. Characterization of Ni(OH)_2_ Hexagonal Nanoplates

**Figure 2:**
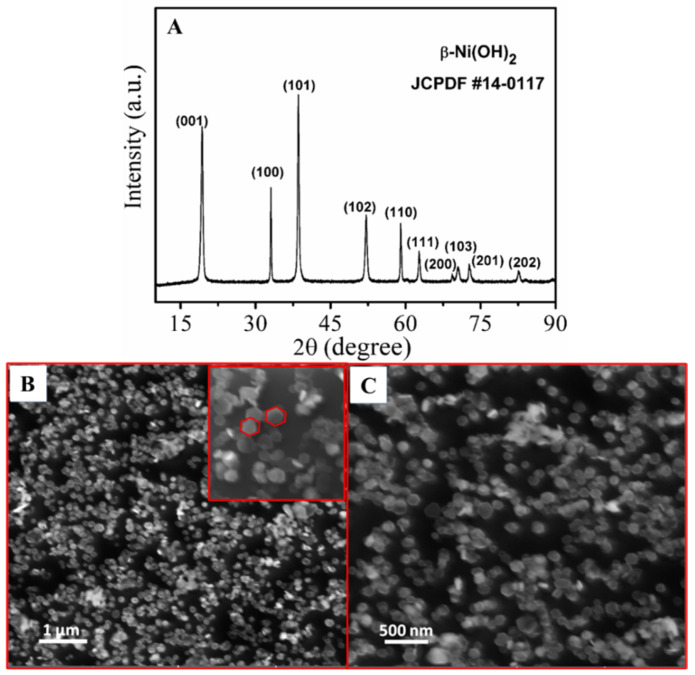
(**A**) XRD patterns of synthesized β-Ni(OH)_2_ nanoplates; (**B**,**C**) FESEM images of β-Ni(OH)_2_ nanoparticles at different magnifications.

The synthesized Ni(OH)_2_ hexagonal nanoplates were subjected to XRD analysis. Figure 2A illustrates the XRD pattern of Ni(OH)_2_ nanoplates. The Ni(OH)_2_ patterns resembled hexagonal-phase -Ni(OH)_2_ (JCPDS No #14-0117) with its associated planes, and no other impurity peaks were observed in the XRD patterns, implying that pure -Ni(OH)_2_ was formed during the synthesis process. All of the diffraction lines were narrow and sharp, indicating an exceptionally crystalline structure for the Ni(OH)_2_ nanoplates. There are no additional peaks due to impurities, indicating that Ni(OH)_2_ crystals are successfully synthesized. 

The surface morphologies of Ni(OH)_2_ nanoplates were investigated using field-emission-scanning electron microscopy (FESEM). A series of FESEM images of β-Ni(OH)_2_ at various magnifications are shown in Figure 2B,C. In this experiment, it was clearly demonstrated that the prepared nanoparticles of β-Ni(OH)_2_ had a regular, hexagonal, plate-like structure with a 100–200 nm size and were uniformly distributed throughout the sample without any aggregation of particles. It could also be inferred that the formed β-Ni(OH)_2_ had a high width-to-thickness ratio, which was responsible for the high electron transfer rate in in electrochemical reactions.

**Figure 3:**
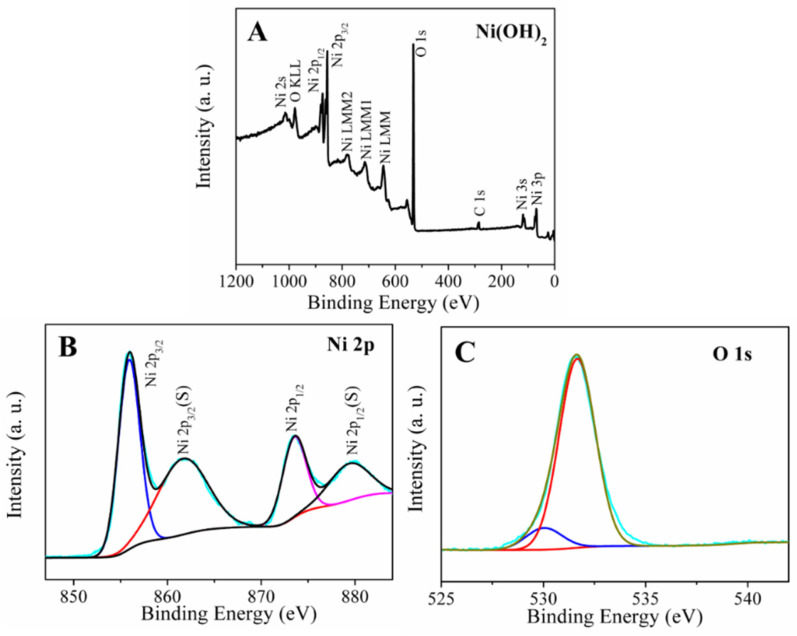
XPS analysis (**A**) The survey scan spectra of β-Ni(OH)_2_ hexagonal nanoplates and the deconvoluted high-resolution XPS spectra of (**B**) Ni 2p and (**C**) O 1s in β-Ni(OH)_2_.

X-ray photoelectron spectroscopy (XPS) was conducted for a better understanding of the material surface chemistry of the Ni(OH)_2_ hexagonal nanoplates. The XPS analysis in the 0–1200 eV energy range was used to confirm the presence of Ni(OH)_2_ nanoplates in the sample (Figure 3). It can be seen in the survey spectrum (Figure 3A), which indicates the occurrence of nickel and oxygen elements in the Ni(OH)_2_ sample. The two representative peaks of Ni 2p3/2 and Ni 2p1/2, which are situated at 855.4 and 873.1 eV, respectively, are visible in the refined de-convolution, accompanied by a suitable spectrum of Ni 2p. The high-resolution, de-convoluted spectrum is accompanied by a suitable spectrum of Ni 2p with a spin-energy difference of about 17.7 eV between Ni 2p3/2 and Ni 2p1/2, corresponding to their respective hydroxide phases and confirming the formation of Ni(OH)_2_. It is possible to see several additional lines along with the Ni 2p1/2 and Ni 2p3/2 signals, which have been identified as satellite peaks (879.2 eV and 861.4 eV). Furthermore, O 1s peak at 531.3 eV in Figure 3C also confirms the presence of Ni-OH bonds in the Ni(OH)_2_ [25].

### 3.2. Electrochemical Activity of NH-HNP-Chit/SPCE

**Figure 4:**
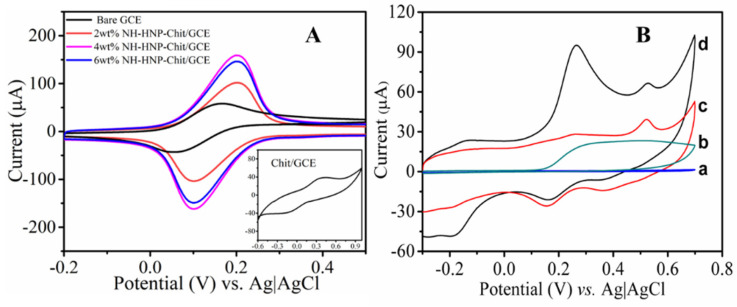
(**A**) Cyclic voltammogram recorded at the bare SPCE, NH-HNP-Chit/SPCE with different Ni(OH)_2_ amounts, and Chit/SPCE (inset) in 1 mM K_3_[Fe(CN)_6_] + 0.1 M KCl. (**B**) Cyclic voltammogram recorded at bare SPCE (a, b) and NH-HNP-Chit/SPCE (c, d) in the presence (b, d) and absence (a, c) of 0.5 mM 5-HT in 0.1 M PBS (pH 7.4). Scan rate of 100 mV/s.

Electrochemical interfacial properties of the modified and unmodified SPCE sensors were characterized using cyclic voltammetry (CV) studies in a ferricyanide redox probe test solution by varying the amounts of the Ni(OH)_2_/chitosan layer. Figure 4A displays the cyclic voltammograms of 2, 4, and 6 wt% NH-HNP-Chit/SPCE, as well as chit/SPCE and bare/SPCE in 1mM K_3_[Fe(CN)_6_] in 0.1M of KCl-supporting electrolyte, at a scan rate of 0.1 V/s. According to the findings, the NH-HNP-Chit/SPCE electrode with a composition of 4 wt% provides the highest level of electrochemical activity when compared to all other electrodes. The extraordinary performance displayed by the 4 wt% NH-HNP-Chit/SPCE is most likely due to its sufficient thickness and the availability of the NH-HNP redox mediator in the chitosan matrix on the surface of the SPCE, which provides maximal current response via the electrochemical reaction of K_3_[Fe(CN)_6_], hence producing maximum activity. From here onwards, the optimized NH-HNP-Chit/SPCE containing 4 wt% NH-HNP was used for 5-HT detection and is indicated as NH-HNP-Chit/SPCE.

Additionally, cyclic voltametric studies were carried out on an optimized NH-HNP-Chit/SPCE electrode in addition to an unmodified electrode in order to develop a better understanding of the electrochemical behavior of 5-HT. The cyclic voltammograms in Figure 4B show the electrochemical oxidation profile of 5-HT in the PBS (pH 7.4) on NH-HNP-Chit/SPCE at the scan rate of 0.1 V/s. The irreversible oxidation peak of 5-HT was observed at +350 mV in both the NH-HNP-Chit/SPCE and bare SPCE, but not in the blank PBS. The peak current due to the electrochemical oxidation of 5-HT is significantly high when the NH-HNP-Chit/SPCE was compared to the bare SPCE; this indicates that the oxidation process of 5-HT was extremely efficient at the NH-HNP-Chit/SPCE. At the same time, 5-HT showed a poor identification peak when subjected to the bare SPCE. The significant increase in oxidation currents at the NH-HNP-Chit/SPCE modified electrode imply that the Ni(OH)_2_ nanoparticles are attributed to the larger active surface area as well as the greater redox nature of the NH-HNP modified electrode, which is consistent with the previous findings. It has been demonstrated that the oxidation of 5-HT is catalyzed by the NH-HNP nanocomposite, resulting in enhanced oxidation current with a slight lowering of oxidation overpotentials after its incorporation. The aforementioned findings may have contributed to the fact that the 5-HT generates a specific and sensitive oxidation peak at the NH-HNP-Chit/SPCE. Further, the electrochemical oxidation mechanism of 5-HT was established based on its reaction behavior when the potential scan rate was varied.

**Figure 5:**
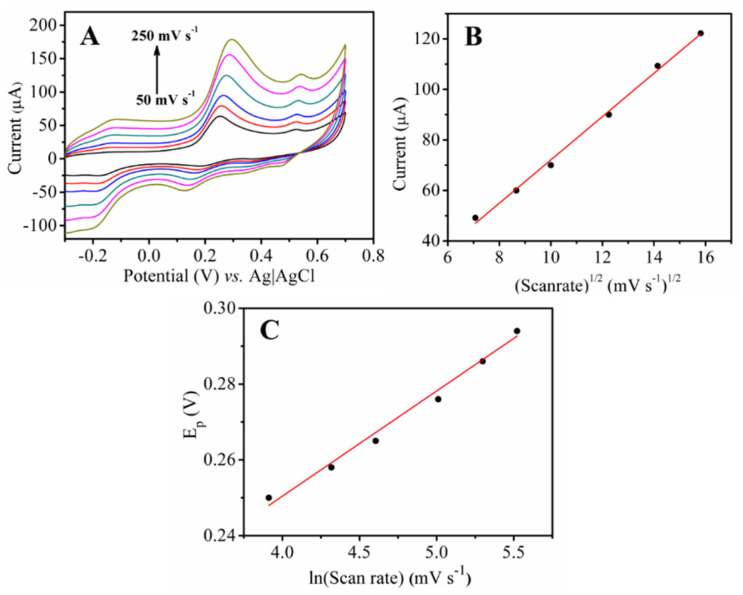
(**A**) Cyclic voltammogram of 0.5 mM 5-HT in 0.1 M PBS (pH 7.4) at the NH-HNP-Chit/SPCE with different scan rates (25 to 250 mV/s). (**B**) Plot of peak current vs. the square root of the scan rate. (**C**) Plot of peak potential vs. ln(scan rate).

**Figure 6:**
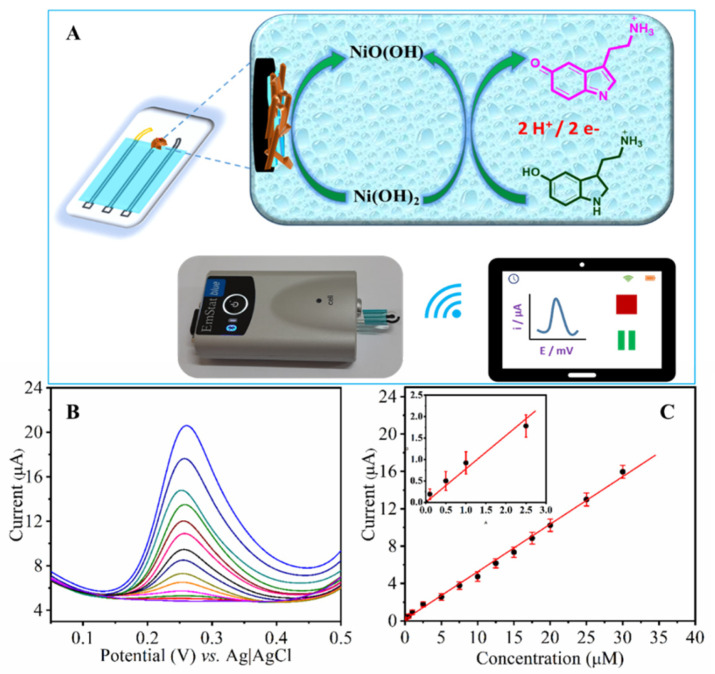
(**A**) Schematic representation of the electrochemical oxidation mechanism of 5-HT at the NH-HNP-Chit/SPCE and data collection with a portable, wireless potentiostat. (**B**) DPV of 5-HT at the NH-HNP-Chit/SPCE in a 0.1 M phosphate buffer solution (pH 7.4) at different concentrations (0.1–30 µM). (**C**). The plot of peak current against the concentration of 5-HT.

The effect of scan rate on the oxidation responses of 5-HT at the NH-HNP-Chit/SPCE was investigated using CV measurements in the scan rate range of 50 to 250 mVs^−1^ (Figure 5). The oxidation peak currents increased steadily as the scan rate increased in the range of 50 to 250 mV s^−1^ (Figure 5A). Peak current values were plotted against the square root of the scan rate (ν^1/2^), yielding a linear relationship (Figure 5B). Based on this behavior, the electro-oxidation of 5-HT at the NH-HNP-Chit/SPCE modified electrode appears to be a diffusion-controlled process. A positive change in the anodic peak potential (Ep) was observed as the scan rate was increased (Figure 5A). According to the findings, Ep increased linearly with ln (scan rate) (Figure 5C). Later, Laviron’s equation was employed to calculate the number of electrons [26,27]. As a result, for the completely irreversible electrode phase, the slope of the Ep vs. ln(scan rate) plot, b = RT/αnF, where α is assumed to be 0.5 for irreversible electrode process. The obtained value for *n* was close to two, indicating that the 5-HT oxidation process involves two electrons. Scheme S1 depicts the plausible electrochemical oxidation mechanism of 5-HT (Supplementary Materials) [28,29]. The overall working mechanism of the disposable NH-HNP-Chit/SPCE sensor towards the detection of 5-HT is represented in Figure 6A.

### 3.3. Quantification of 5-HT by DPV

The analytical performance of the fabricated NH-HNP-Chit/SPCE towards the 5-HT was determined using the DPV. It is evident from Figure 6B that the 5-HT exhibits a well-defined voltammogram with an anodic peak at +0.26 V under optimized conditions using the NH-HNP-Chitosan-modified, screen-printed electrode. Figure 6B also shows the DPV response recorded at the NH-HNP-Chit/SPCE at various 5-HT concentrations ranging from 0.1 to 30 µM. The calibration graph obtained from DPV analysis with peak current versus the 5-HT concentration is shown in Figure 6C. The anodic peak current has been increased linearly as serotonin concentration increases in above-mentioned concentration region. A low detection limit of 60 nM was obtained by taking the peak current changes of three relative standard deviations, which correlates to the real ranges of 5-HT in healthy human plasma [30]. This suggests that the proposed sensor device may be capable of measuring 5-HT from physiological samples. Further, the results obtained from the present sensor system towards the quantification of 5-HT have been compared with those previously reported various electrochemical sensor materials (Table S2, Supplementary Materials) [31,32,33,34,35,36,37,38,39,40,41]. The present NH-HNP-Chit/SPCE outperforms most of the reported sensing materials for the detection of 5-HT.

### 3.4. Interference Analysis

**Figure 7:**
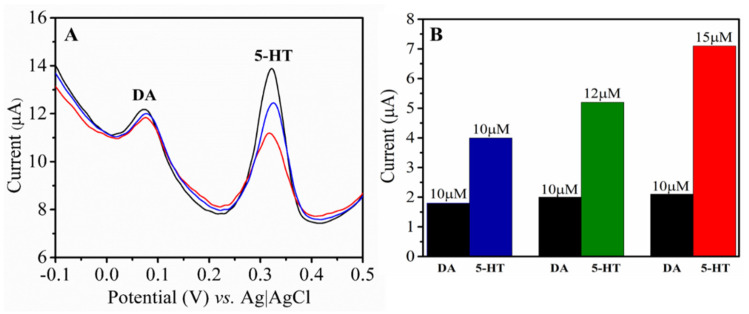
(**A**) DPVs recorded at the NH-HNP-Chit/SPCE in 5-HT at different concentrations (10, 12 and 15 µM) and in the presence of DA (10 µM). (**B**) Bar representation of the NH-HNP-Chit/SPCE response for different concentrations of 5-HT in the presence of DA (10 µM).

The selectivity of the proposed sensor was tested by DPV at the NH-HNP-Chit/SPCE-modified electrode in the presence of another major neurotransmitter, dopamine (DA), along with the target analyte. Figure 7A depicts the DPVs at the NH-HNP-Chit/SPCE in the PBS buffer (pH 7.4) containing different amounts of 5-HT (10, 15, and 20 µM) along with 10 µM DA. The DA and 5-HT peaks were discovered at distinct oxidation potentials, and it was discovered that, in the presence of dopamine, the 5-HT peak position was slightly displaced towards a higher potential (Figure 7A). Despite the presence of DA as an interferent, the current response to 5-HT increased with increasing concentration was also in good agreement with the above calibration plot (Figure 7B). Surprisingly, even at further increased DA concentrations, there no noticeable change in the 5-HT response was detected. Based on these observations, it is proposed that the current sensor system could detect 5-HT selectively, even in the presence of a substantial, active physiological interference neurotransmitter molecule. 

### 3.5. Reproducibility, Reusability and Long-Term Stability

The reproducibility, stability, and reusability of the current sensor system for the detection of 5-HT using DPV measurements were investigated. Over the course of a week, the DPVs of 10 µM 5-HT were reported at the same NH-HNP-Chit/SPCE electrode, and it was discovered that the voltametric current response of 5-HT only decreased by 3%. These findings strongly support that the established sensor system is very stable and reusable even when stored in ambient laboratory conditions. The fabricated electrodes reproducibility was tested by recording the DPV scans for five NH-HNP-Chit/SPCE electrodes prepared separately in the presence of 10 µM 5-HT. At these electrodes, the DPV peak currents of five individual NH-HNP-Chit/SPCE electrodes differed just by 3.2% (n = 5). It is clear that the NH-HNP-Chit/SPCE nanocomposite electrode has excellent reproducibility when it is aimed at detecting 5-HT. After taking the findings into consideration, it may be concluded that the repeatability and reusability of the present sensor system are quite satisfactory. 

### 3.6. Real Sample Analysis (Synthetic Urine and Saliva)

The constructed sensor was then tested for the detection of 5-HT in laboratory-prepared artificial urine and saliva solutions. We employed the standard addition method for the quantification of 5-HT in the synthetic urine and saliva samples to test the fabricated NH-HNP-Chit/SPCE nanocomposite electrode under physiological conditions. The synthetic urine and saliva solutions were prepared as described in the literature [42,43,44], and lists all of the compounds required, and their concentrations provided in Table S1. Furthermore, in each sample, we examined three concentrations: one close to LOD (0.5 µM), and another two in the linear range (2 and 5 µM). Table 1 shows the recovery of 5-HT from artificial urine and saliva samples at varied concentrations which ranged from 94.5% to 104.0%. The results reveal that the constructed NH-HNP-Chit/SPCE sensor could be a suitable candidate for the selective detection of 5-HT from physiological samples.

## 4. Conclusions

For the measurement of serotonin at a physiologic pH, a nano-level detectable and disposable screen-printed electrochemical sensor was designed. The SPCE was modified by casting it with a NH-HNP-Chit mixture, and the resultant electrode had high electro-oxidation capabilities for 5-HT. At 0.35 V, the modified NH-HNP-Chit/SPCE electrode displays a well-defined serotonin oxidative response. The use of the NH-HNP-Chit/SPCE electrode enhanced the oxidation peak current of the 5-HT molecule when compared to use of the SPCE in PBS (pH 7.4), and the DPV peak potential of 5-HT was well separated. The detection limit of the NH-HNP-Chit/SPCE for 5-HT has been achieved as low as 60 nM. The designed NH-HNP-Chit/SPCE-composite-film electrode functions as an effective electron-promoter and bridges the target and the electro-active surface to enhance the current response of serotonin. The nanocomposite coating on the SPCE surface was very stable and resistant to numerous analyses over a long period of time due to the stable, film-forming behavior of chitosan biopolymer. The easy manufacturing process, low cost, long-term stability, wide linear range, and low detection limit indicate that this disposable NH-HNP-Chit/SPCE could be a potential candidate for in vitro quantification of serotonin from physiological fluids, a depression biomarker.

## Figures and Tables

**Table 1:** Determination of 5-HT in artificial urine and saliva samples using NH-HNP-Chit/SPCE.

Sample	5-HT	^a^ Found	Average Recovery (%)	^a^ RSD
	(×10^−6^ M)	(×10^−6^ M)		(%)
Urine Sample	0.5	0.52	104.0	0.9
	2	1.97	98.5	1.4
	5	4.92	98.4	2.7
Saliva samples	0.5	0.48	96.0	1.2
	2	1.89	94.5	0.8
	5	4.82	96.4	1.9

^a^ Mean value of six measurements.

## Data Availability

Not applicable.

## Supplementary Materials

The following supporting information can be downloaded at: https://www.mdpi.com/article/10.3390/mi14010146/s1, Scheme S1: Plausible electrochemical oxidation reaction mechanism of 5-HT; Table S1: Composition of prepared artificial urine and saliva samples; Table S2: Comparison of working concentration range and detection limit of 5-HT by NH-HNP-Chit/SPCE with recently reported methods.
