# Supplementary material for: A Disposable Screen Printed Electrodes with Hexagonal Ni(OH)2 Nanoplates Embedded Chitosan Layer for the Detection of Depression Biomarker
Source: Micromachines (Basel). 2023 Jan 5;14(1):146. doi: 10.3390/mi14010146 (PMC9861775; doi:10.3390/mi14010146)
Supplement: Supplementary file 1 [file micromachines-14-00146-s001.zip › micromachines-2103353-supplementary.pdf]

micromachines

Supplementary Materials

A Disposable Screen Printed Electrodes with Hexagonal Ni(OH)2 Nanoplates Embedded Chitosan Layer for the Detection of Depression Biomarker

Satyanarayana Moru [1,*], Venishetty Sunil Kumar [2], Shekar Kummari [3] and Kotagiri Yugender Goud [3,4]

[1] Department of Chemistry, School of Advanced Sciences, VIT-AP University, Amaravati 522237, India
[2] Department of Physical Sciences, Kakatiya Institute of Technology & Science, Warangal 506015, India
[3] Department of Chemistry, Indian Institute of Technology Palakkad, Palakkad 678 557, India
[4] Institute of Nanobiotechnology, Johns Hopkins University, Baltimore, MD 21218, USA
* Correspondence: dr.morusatyanarayana@gmail.com or satyanarayana.moru@vitap.ac.in; Tel.: +91-9959704030

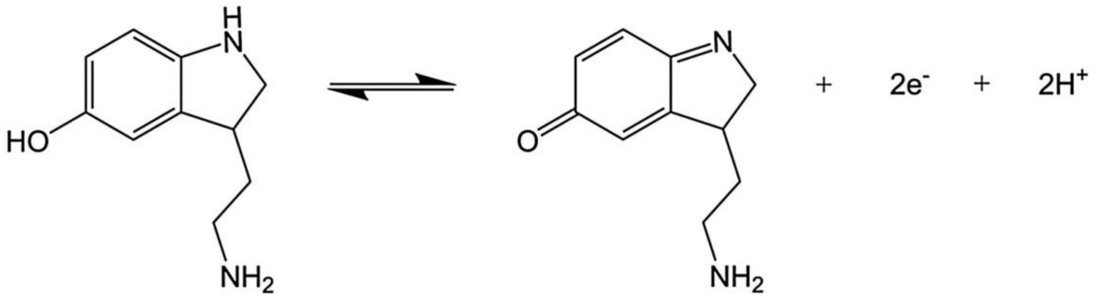

Scheme 1. Plausible electrochemical oxidation reaction mechanism of 5-HT.

Table S1. Composition of prepared artificial urine and saliva samples.

Chemical component	Synthetic Urine (g L^{-1})	Synthetic Saliva (g L^{-1})
$CaCl_2{\cdot}2H2O$	1.12	0.81
NaCl	2.92	0.40
Na_2SO_4	2.24	–
KH_2PO_4	1.40	0.78
KCl	1.60	0.43
NH_4Cl	1.00	–
Urea	25.0	1.00
Creatinine	1.10	–

Table S2. Comparison of working concentration range and detection limit of 5-HT by NH-HNP-Chit/SPCE with recently reported methods.

**Electrode**	**Detection Method**	**Linear Range**	**Limit of detection**	**Reference**
MCM-41-COOH/Au@nano-CILPE	SWV	0.2 – 20 μM	0.1 μM	[S1]
Pt/MWCNT/PPy/AgNPs	DPV	0.50 – 5.0 μmol L^{-1}	0.15 μmol L^{-1}	[S2]
DDF-CNT-TiO_2/IL/GC	DPV	1.0 – 650.0 μM	0.154 μM	[S3]

GCE/MWCNT-NiO	SWV		118 nM	
GCE/MWCNT-ZnO	SWV	5.98×10^{-3} - 62.8 μM	129 nM	[S4]
GCE/MWCNT-Fe3O4	SWV		166 nM	
PEDOT:PSS/TPyP– 3IP/FTO	CV	-	0.23 μM	[S5]
NiO/CNT/PEDOT/GCE	DPV	0.3 – 35 μM	0.063 μM	[S6]
MWCNT/GC	DPV	5 – 210 μM	460 nmol L^{-1}	[S7]
IL–DC–CNT/GC	DPV	5.0 – 900.0 μM	2 μM	[S8]
polymelamine/EPPGS	SWV	0.1 – 100 μM	30 nM	[S9]
G-g-PLA-Pd/GCE	amperometric	0.1–100.0 μM	0.08 μM	[S10]
MWCNT-Chit/GCE	DPV	0.05–16 μM	50 nM	[S11]
NH-HNP-Chit/SPCE	DPV	0.1 – 30 μM	60 nM	This work

MCM-41-COOH-carboxyl-functionalized mesoporous molecular sieve; ILPE-ionic liquid paste electrode; MWCNT- multi-walled carbon nanotubes; PPy- polypyrrole; AgNPs- silver nanoparticles; DDF- 9-(1,3-dithiolan-2-yl)-6,7-dihydroxy-3,3-dimethyl-3,4-dihydrodibenzo[b,d]furan-1(2H)-one; IL- 1-butyl-3-methylimidazolium tetrafluoroborate; (TPyP)-tetrakis(4-pyridyl)-21H,23H-porphyrin; PEDOT-poly(3,4-ethylenedioxythiophene); IL-ionic liquid; EPPGS-edge plane pyrolytic graphite sensor; PLA- poly(lactic acid).

References

S1. Li, Y.; Ji, Y.; Ren, B.; Jia, L.; Ma, G.; Liu, X. Carboxyl-functionalized mesoporous molecular sieve/colloidal gold modified nano-carbon ionic liquid paste electrode for electrochemical determination of serotonin. *Mater. Res. Bull.* **2019**, *109*, 240–245. https://doi.org/10.1016/j.materresbull.2018.10.002.

S2. Cesarino, I.; Galesco, H.V.; Machado, S.A. Determination of serotonin on platinum electrode modified with carbon nanotubes/polypyrrole/silver nanoparticles nanohybrid. *Mater. Sci. Eng. C* **2014**, *40*, 49–54. https://doi.org/10.1016/j.msec.2014.03.030.

S3. Mazloum-Ardakani, M.; Khoshroo, A. Electrocatalytic properties of functionalized carbon nanotubes with titanium dioxide and benzofuran derivative/ionic liquid for simultaneous determination of isoproterenol and serotonin. *Electrochim. Acta* **2014**, *130*, 634–641. https://doi.org/10.1016/j.electacta.2014.03.063.

S4. Fayemi, O.E.; Adekunle, A.S.; Ebenso, E.E. Electrochemical determination of serotonin in urine samples based on metal oxide nanoparticles/MWCNT on modified glassy carbon electrode. *Sens. Bio-Sens. Res.* **2017**, *13*, 17–27. https://doi.org/10.1016/j.sbsr.2017.01.005.

S5. Song, M.-J.; Kim, S.; Min, N.K.; Jin, J.-H. Electrochemical serotonin monitoring of poly(ethylenedioxythiophene):poly(sodium 4-styrenesulfonate)-modified fluorine-doped tin oxide by predeposition of self-assembled 4-pyridylporphyrin. *Biosens. Bioelectron.* **2014**, *52*, 411–416. https://doi.org/10.1016/j.bios.2013.08.040.

S6. Sun, D.; Li, H.; Li, M.; Li, C.; Dai, H.; Sun, D.; Yang, B. Electrodeposition synthesis of a NiO/CNT/PEDOT composite for simultaneous detection of dopamine, serotonin, and tryptophan. *Sensors Actuators B Chem.* **2018**, *259*, 433–442. https://doi.org/10.1016/j.snb.2017.12.037.

S7. Bonetto, M.C.; Muñoz, F.F.; Diz, V.E.; Sacco, N.J.; Cortón, E. Fused and unzipped carbon nanotubes, electrochemically treated, for selective determination of dopamine and serotonin. *Electrochim. Acta* **2018**, *283*, 338–348. https://doi.org/10.1016/j.electacta.2018.06.179.

S8. Mazloum-Ardakani, M.; Khoshroo, A. High sensitive sensor based on functionalized carbon nanotube/ionic liquid nanocomposite for simultaneous determination of norepinephrine and serotonin. *J. Electroanal. Chem.* **2014**, *717–718*, 17–23. https://doi.org/10.1016/j.jelechem.2013.12.034.

S9. Gupta, P.; Goyal, R.N. Polymelamine modified edge plane pyrolytic graphite sensor for the electrochemical assay of serotonin. *Talanta* **2014**, *120*, 17–22. https://doi.org/10.1016/j.talanta.2013.11.075.

S10. Han, H.S.; You, J.-M.; Jeong, H.; Jeon, S. Synthesis of graphene oxide grafted poly(lactic acid) with palladium nanoparticles and its application to serotonin sensing. *Appl. Surf. Sci.* **2013**, *284*, 438–445. https://doi.org/10.1016/j.apsusc.2013.07.116.

S11. Satyanarayana, M.; Koteshwara Reddy, K.; Vengatajalabathy Gobi, K. Nanobiocomposite Based Electrochemical Sensor for Sensitive Determination of Serotonin in Presence of Dopamine, Ascorbic Acid and Uric Acid In Vitro. *Electroanalysis* **2014**, *26*, 2365–2372. https://doi.org/10.1002/elan.201400243
